# The influence of students’ prior clinical skills and context characteristics on mini-CEX scores in clerkships – a multilevel analysis

**DOI:** 10.1186/s12909-015-0490-3

**Published:** 2015-11-25

**Authors:** Anja Rogausch, Christine Beyeler, Stephanie Montagne, Patrick Jucker-Kupper, Christoph Berendonk, Sören Huwendiek, Armin Gemperli, Wolfgang Himmel

**Affiliations:** 1 Department of Assessment and Evaluation, Institute of Medical Education, University of Bern, Bern, Switzerland; 2 Department of Health Sciences and Health Policy, University of Lucerne, Lucerne, Switzerland; 3 Swiss Paraplegic Research Nottwil, Nottwil, Switzerland; 4 Department of General Practice, University Medical Center, Göttingen, Germany; 5 Clinic Sonnenhalde, Riehen, Switzerland

**Keywords:** Clinical Competence, Educational measurement, Education, Medical, Undergraduate, Task Performance and analysis, Workplace, Clinical clerkship, Psychometrics

## Abstract

**Background:**

In contrast to objective structured clinical examinations (OSCEs), mini-clinical evaluation exercises (mini-CEXs) take place at the clinical workplace. As both mini-CEXs and OSCEs assess clinical skills, but within different contexts, this study aims at analyzing to which degree students’ mini-CEX scores can be predicted by their recent OSCE scores and/or context characteristics.

**Methods:**

Medical students participated in an end of Year 3 OSCE and in 11 mini-CEXs during 5 different clerkships of Year 4. The students’ mean scores of 9 clinical skills OSCE stations and mean ‘overall’ and ‘domain’ mini-CEX scores, averaged over all mini-CEXs of each student were computed. Linear regression analyses including random effects were used to predict mini-CEX scores by OSCE performance and characteristics of clinics, trainers, students and assessments.

**Results:**

A total of 512 trainers in 45 clinics provided 1783 mini-CEX ratings for 165 students; OSCE results were available for 144 students (87 %). Most influential for the prediction of ‘overall’ mini-CEX scores was the trainers’ clinical position with a regression coefficient of 0.55 (95 %-CI: 0.26–0.84; *p* < .001) for residents compared to heads of department. Highly complex tasks and assessments taking place in large clinics significantly enhanced ‘overall’ mini-CEX scores, too. In contrast, high OSCE performance did not significantly increase ‘overall’ mini-CEX scores.

**Conclusion:**

In our study, Mini-CEX scores depended rather on context characteristics than on students’ clinical skills as demonstrated in an OSCE. Ways are discussed which focus on either to enhance the scores’ validity or to use narrative comments only.

**Electronic supplementary material:**

The online version of this article (doi:10.1186/s12909-015-0490-3) contains supplementary material, which is available to authorized users.

## Background

Learner assessment in medical education is becoming increasingly oriented towards defined outcomes, including the adequate application of skills and knowledge in the clinical setting [1–3]. Workplace-based assessments (WBAs) have emerged as an important element in this development, aiming to assess on the highest level of Miller’s pyramid, the ‘does’ level [4, 5]. They complement other assessment methods, such as objective structured clinical examinations (OSCEs), which also assess clinical skills but on Miller’s ‘shows how’ level within a more standardized setting [6].

Both OSCEs and WBAs include the observation of a learner by a trained teacher, structured by a rating form. While OSCEs are based on different pre-defined stations, WBAs – such as the mini-clinical evaluation exercises (mini-CEXs) [7, 8] – comprise less pre-defined encounters in daily clinical practice. Nevertheless, both in OSCEs and mini-CEXs, multiple scores averaged across these different encounters/stations and including different cases, patients and raters, should represent a valid picture of a learner’s clinical skills [3].

It is well documented that OSCEs can provide reliable and valid results [9–12]. Similarly, several studies reported evidence for the reliability and validity of mini-CEXs [13–17]. However, in their review of WBA tools, Pelgrim et al. emphasized that ‘due to their quite subjective nature, instruments like the mini-CEX may actually be quite vulnerable to bias’ [16]. In everyday clinical applications of WBAs, the assessment quality depends on the raters who are using the tool, and potentially also on context characteristics which influence the raters’ information processing [18–21]. These context characteristics are summarized in the model of the performance assessment process, developed by DeNisi and adapted by Govaerts [18, 22]. It comprises cues related to (1) the learners’ performance, (2) the raters, (3) the assessment design and (4) the organizational environment which all influence trainers’ ratings in a certain way [18, 22].

It is therefore not surprising that WBA scores from standardized settings without personal interaction with the learner (e.g. judgment of videotaped performance) often differ from WBA scores in clinical workplace settings: In everyday clinical workplace settings, trainers – serving as raters in WBA – are prone to halo effects, range restriction and leniency [1, 15, 23–27] and do not differentiate between performance levels or between performance dimensions [1, 24, 28]. In contrast, studies from standardized WBA settings which share some similarities with OSCEs (i.e. rating of performance of unfamiliar students, no personal feedback) showed that, in principle, trainers have the ability to apply WBA tools such as mini-CEXs precisely to discriminate different levels of learners’ performance [29, 30] as well as different performance dimensions [31].

Little is known about how strong the influence of context variables at the workplace is in relation to a student’s clinical skills, which can be assessed in a quite reliable and unbiased way by means of an OSCE [27, 32]. Thus, the aim of this study was to determine the relationship between a student’s prior performance in a standardized setting and his or her subsequent ratings on the workplace, while taking variables from the social context into account. In particular, we analyzed clinic characteristics (clinic size, which is reported to be associated with different workplace conditions [32]), trainers’ clinical position (which is reported to be associated with expertise and rater stringency [33] and task complexity (which is reported to influence rater information processing as well) [8].

We hypothesized that the students’ clinical skills are the strongest predictor for their subsequent mini-CEX scores during clerkships, and that variables relating to the context play a minor role. This analysis allows a more detailed view on the cues impacting WBA scores or, in other words, what WBA scores really reflect in our setting.

## Methods

### Setting

At the end of Year 3 of the 6 year curriculum, all medical students took part in a mandatory OSCE six months before beginning the subsequent clerkships in Year 4. During their clerkships in surgery, gynecology, internal medicine, pediatrics and psychiatry (each of 4 weeks duration), all Year 4 medical students at the University of Bern have to perform a specified number of one to three mini-CEXs per discipline in addition to a variable number of Direct Observation of Procedural Skills (DOPS), i.e. 1 Mini-CEX and 2 DOPS in surgery, 2 Mini-CEX and 1 DOPS in gynecology, 3 Mini-CEX in pediatrics, 2 Mini-CEX and 1 DOPS in internal medicine, 3 Mini-CEX in psychiatry. Clerkships took place in clinics at the University Medical Centre or in affiliated teaching hospitals.

### Instruments and outcomes

#### Objective structured clinical examination

The OSCE included 9 clinical skills stations (gynecology, geriatrics, primary care, neurology, musculoskeletal system, pediatrics, psychiatry, ophthalmology, ear/nose/throat [ENT]). OSCE raters attended an 1-h training workshop where the principles of OSCE assessment were taught. Students were randomly assigned to two equivalent OSCE tracks on two subsequent days, comprising comparable clinical tasks related to history-taking and physical examination. The reliabilities of the OSCE tracks were 0.78 and 0.74, respectively (Cronbach’s Alpha based on 170 and 168 checklist criteria, respectively).

#### Mini-clinical evaluation exercises

**Fig. 1:**
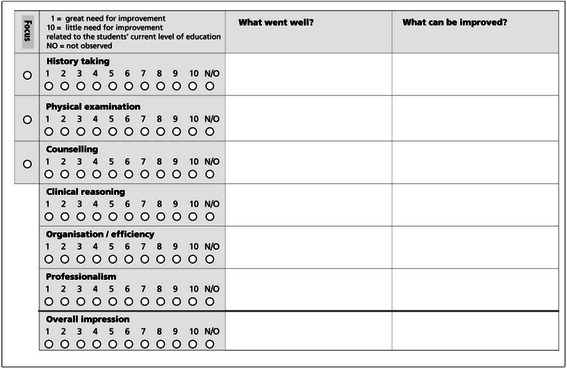
Example of the mini-CEX checklist

Mini-CEX cover the following domains: history-taking, physical examination (for psychiatry: psychiatric examination), counselling, clinical judgment, organization/efficiency and professionalism. Additionally, a global score regarding the ‘overall’ impression of a student’s performance can be assigned. As is the case for many applications of mini-CEX, we introduced minor adaptations of the rating scale and form, based on the original mini-CEX form developed by the American Board of Internal Medicine [34]: Trainers were asked to rate the students’ performance on a 10-point mini-CEX rating scale, ranging from 1 to 10 (i.e. great to little need for improvement; related to the current level of education; depicted in Fig. 1). This adaptation of the original rating scale should prevent range restriction and grade inflation which we witnessed using the original scale during the pilot implementation in Switzerland [35].

Interactive workshops (approx. 2.5 h) and written material regarding the purpose and procedures of mini-CEXs were offered for all trainers responsible for the clerkships. Additionally, all trainers had access to all course material, including videos of student-patient interactions for practical in-house feedback training. This training approach is typical for the clinical context, where time for training competes with time for patient management.

Within this study, the alignment between identified learning needs during a mini-CEX and the resulting learning goals was analyzed separately [36].

### Analyses

As preparatory work, first descriptive analyses regarding assessment and score characteristics were performed, in order to assess (1) whether grade inflation was given in the data, (2) whether averaging mini-CEX domain scores was justified and (3) to describe the correlation between mini-CEX and OSCE scores. Afterwards, the relationship between mini-CEX and OSCE scores was analyzed in more detail, in taking variables from the social context into account (linear regression analyses in a multilevel design).

#### Characteristics of assessments and mini-CEX scores

We first focused on two parts of the mini-CEX: ‘overall’ and ‘domain’ scores (i.e. history-taking, physical examination etc.). As mini-CEX scores from more than 7 encounters were reported to be sufficient to produce defensible reliability [23], we used mini-CEX scores averaged from all 11 mini-CEXs of each student.

**Fig. 2:**
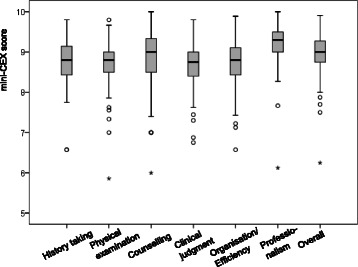
Boxplot of mean trainers’ scores regarding each of domains and ‘overall’ scores of the mini-CEX

Thus, mean *‘overall’* scores represent the averaged ‘overall’ scores from all mini-CEXs of each student (Cronbach’s Alpha = .70): As most students performed 11 mini-CEX, mean *‘overall’* scores were computed by averaging all single ‘overall’ scores of these 11 mini-CEX (depicted in Fig. 2).

**Fig. 3:**
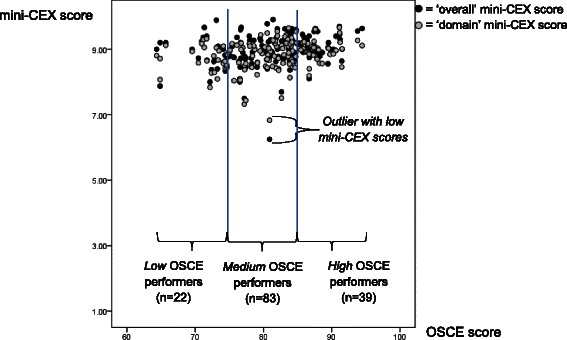
Mean scores of the OSCE in relation to ‘overall’ (black bullets) and ‘domain’ mini-CEX scores (grey bullets)

For mean *‘domain’* scores, the mean of the scores related to each of the 6 domains was computed, averaged over the 11 mini-CEXs of each student (i.e. history-taking, physical examination etc., as depicted in Fig. 2). As raters typically do not differentiate between the six mini-CEX domains, but these 6 ‘domain’ mini-CEX scores represent a single global dimension of clinical competence [28], which was supported by our own analyses (Cronbach’s Alpha = .86; data not shown), averaging all 6 ‘domain’ scores of the 11 mini-CEX was justified. The resulting mean ‘overall’ and ‘domain’ mini-CEX scores for each student (in relation to their mean OSCE scores) will be depicted in Fig. 3.

#### Correlation between mini-CEX and OSCE scores

For 144 of the 165 students analyzed in this study, pseudonymised OSCE data were available (the remaining students had taken the OSCE the previous year or were students from another university). We computed mean scores for each student based on all checklist criteria of the 9 clinical skills stations (170 and 168 checklist criteria of the two equivalent OSCE tracks, respectively) as well as based on the 5 stations which were directly reflected in the clerkships (i.e. gynecology, pediatrics and psychiatry, as well as geriatrics and primary care which were reflected by the clerkships in internal medicine). Subsequently, Pearson correlations were calculated between mean OSCE scores (first based on the checklist criteria of all stations and second based on the restricted number of stations) and mean ‘overall’ mini-CEX scores, both of which were normally distributed.

#### Prediction of mini-CEX scores

Linear regression analyses were performed in order to separately predict ‘overall’ and ‘mean domain’ mini-CEX scores as criteria (with the latter representing the mean of the 6 mini-CEX domain scores). Using ‘Proc mixed’ in SAS, we embedded the analysis in a multilevel design by introducing random effects to adjust for the multiple observations within specialties, clinics, trainers and students and to capture within-cluster associations. The fixed effect estimates for the predictor variables were expressed on the same scale as the mini-CEX scores and interpreted as absolute changes conditional on same levels of the random effects. Regression analyses were performed in two steps:

1. First, separate regression models with single predictors were performed to analyze the association between the above-mentioned criteria and the following potential predictors:
  *Clinic characteristics*
    Clinic size: clinics were divided into three categories depending on the number of students completing a clerkship in this clinic per year (<15 students/year = ‘small’ clinic; 16–30 students/year = ‘medium-sized’ clinic; > 30 students/year = ‘large’ clinic).
  *Trainer and student characteristics*
    Trainers’ clinical position (i.e. resident, senior physician, head of department),
    Students’ gender,
    Students’ OSCE performance (three groups of increasing OSCE results: low, medium and high OSCE performers, each of identical width; see Fig. 3).
  *Assessment characteristics (mini-CEX)*
    Duration of observation (lower vs. greater than median, i.e. 15 min),
    Task complexity (low, medium, high),
    First, second, third mini-CEX per clerkship.
2. All predictors showing an association (*p* < 0.20, likelihood ratio test) with the outcome in the separate analyses were retained in the final multifactorial model, with a significance level of *p* < .05 (two-sided). The relative influence of the four random effects on the scores, i.e. specialties, clinics, trainers and students, was calculated by the covariance parameter estimates. Standard error in regression coefficients have been corrected for uncertainty in model covariance and bias by exerting denominator degrees of freedom according to Kenward and Roger. A visual inspection of residuals was conducted to verify the model assumptions, such as randomness and normal distribution of residuals and identification of outliers. All analyses were performed using SAS 9.3 for Windows (SAS Institute, Inc., Cary, NC, USA). An example of the SAS Statement that we used for the final models can be seen in the Additional file 1.

## Results

### Sample characteristics and general assessments

The data set comprised 1783 mini-CEX ratings (1773 mini-CEX ratings after exclusion of one student with extremely low mini-CEX scores) by 512 trainers (57 % female) for 165 students (58 % female) in 45 clinics. Approximately 92 % of the students submitted the required number of 11 mini-CEXs (14 students submitted 9 mini-CEXs on average; range 5–10 mini-CEXs). Table 1 shows characteristics of clinics, trainers and tasks as well as duration of observation and feedback. One student with extremely low mini-CEX scores (but medium OSCE scores) had been excluded from further statistical analyses, because we wanted to avoid over- or underestimation of the association between mini-CEX and OSCE scores (for transparency, these scores are highlighted in Figs. 2 and 3).

**Table 1:** Characteristics of clinics, trainers, tasks, observation and feedback during mini-clinical evaluation exercises (mini-CEX)

Clinic size	‘small’	29 clinics with *n* = 437 trainers’ ratings^a^
	‘medium-sized’	10 clinics with *n* = 462 trainers’ ratings
	‘large’	6 clinics with *n* = 874 trainers’ ratings
Trainers	residents	54 % of the mini-CEX (*n* = 957)
	senior physicians	36 % of the mini-CEX (*n* = 638)
	heads of department	9 % of the mini-CEX (*n* = 160)
	no information	1 % of the mini-CEX (*n* = 18)
Task complexity	‘low complexity’	9 % of the mini-CEX (*n* = 160)
	‘medium complexity’	65 % of the mini-CEX (*n* = 1152)
	‘high complexity’	17 % of the mini-CEX (*n* = 301)
	no information	9 % of the mini-CEX (*n* = 160)
Duration	of observation	Median = 15 min
	of feedback	Median = 5 min

^a^For a total of 1773 mini-CEX after exclusion of one student with 10 mini-CEX (outlier)

### Mini-CEX scores and their correlation with OSCE scores

High mean scores were found for trainers’ ‘*overall’* scores and for each of the 6 mini-CEX domains (Fig. 2). High scores were already observed for the first Mini-CEXs at the beginning of the clerkships, were observable within each of the clerkship disciplines and, on average, showed no systematic increase during the year (data not shown).

OSCE scores showed only weak correlations with the mean ‘*overall’* mini-CEX scores (*r* = 0.26) and the trainers’ mean ‘*domain’* mini-CEX scores (*r* = 0.27; see Fig. 3). Restricting the OSCE scores to the stations which were reflected in the clerkships (i.e. gynecology, geriatrics, primary care, pediatrics, psychiatry) resulted in equally low correlations with trainers’ mean ‘*overall’* mini-CEX scores (*r* = 0.20) and ‘*domain’* mini-CEX scores (*r* = 0.21).

### Prediction of mini-CEX scores

All mixed linear regressions fit well according to residual diagnostics; the original SAS Outputs can be seen in the Additional file 1. Several predictors were identified as relevant for the prediction of trainers’ ‘*overall’* mini-CEX scores and were included in the multifactorial model (Table 2: right column): Most influential for the prediction of ‘overall’ scores was the trainers’ clinical position, with a regression coefficient of 0.55 (95 %-confidence interval CI: 0.26–0.84; *p* < .001) for residents compared to heads of department. Additionally, highly complex tasks and assessments taking place in large clinics also increased ‘*overall’* scores. In contrast, ‘*overall’* mini-CEX scores for students from the low-performing OSCE group (or medium performers, respectively) were only slightly lower compared to the high-performing OSCE group (regression coefficient −0.15 [95 %-CI: −0.36–0.063] for low vs. high performers, for medium vs. high performers: −0.13 [95 %-CI: −0.28–0.021]; Table 2).

**Table 2:** Estimated regression coefficients for the prediction of trainers’ ‘*overall*‘*mini-CEX scores,* including random effects

	Estimated regression coefficients (and 95 % confidence intervals)	
Predictor	Single predictors	Multifactorial model
Clinic size		
Small vs. large	−0.25* (-0.48, −0.019)	−0.26* (−0.48, −0.039); DF = 19.3
Medium vs. large	−0.25 (−0.53, −0.012)	−0.17 (−0.42, 0.077); DF = 17
Trainers’ function		
Resident vs. head of department	0.54*** (0.28, 0.81)	0.55*** (0.26, 0.84); DF = 452
Senior physician vs. head of department	0.10 (−0.17, 0.37)	0.12 (−0.18, 0.42); DF = 453
Students’ gender		
Male vs. female	−0.09 (−0.21, 0.026)	−0.062 (−0.20, 0.074); DF = 138
Assessment characteristics		
Low vs. high complexity	−0.24* (−**0.43,** −**0.04)**	−0.23* (−**0.44,** −**0.019)**; DF = 1049
Medium vs. high complexity	−0.059 (−0.18, 0.067)	−0.081 (−0.21, 0.052); DF =1015
Students’ clinical skills (OSCE)		
Low vs. high performers	−0.19 (−0.39, 0.014)	−0.15 (−0.36, 0.063); DF = 132
Medium vs. high performers	−0.15 (−0.29, 0.0021)	−0.13 (−0.28, 0.021); DF = 132

Predictors remaining significant in the multifactorial model are reported in **bold**

*DF* degrees of freedom

*p < 0.05

**p < 0.01

***p < 0.001

In terms of ‘*mean domain’* scores, trainers’ clinical position and clinic size were also the most important predictors. In contrast to the prediction of ‘*overall’* scores, task complexity did not play a role (i.e. was not included in the multifactorial model). ‘*Mean* d*omain’* mini-CEX scores of low OSCE performers were −0.13 (95 %-CI: −0.33–0.078) lower than those of high OSCE performers (−0.18; 95 %-CI: −0.33 to −0.038 for medium vs. high OSCE performers; Table 3).

**Table 3:** Estimated regression coefficients for the prediction of trainers’ *mean* ‘*domain*‘*mini-CEX score*s, including random effects

	Estimated regression coefficients (and 95 % confidence intervals)	
Predictor	Single predictors	Multifactorial model
Clinic size		
Small vs. large	−0.25* (−**0.50,** −**0.010)**	−0.27* (−**0.51,** −**.032)**; DF = 20.3
Medium vs. large	−0.24 (−0.52, 0.031)	−.20 (−0.47, .069); DF = 19
Trainers’ function		
Resident vs. head of department	0.59*** (0.34, 0.84)	0.59*** (0.32, 0.87); DF = 444
Senior physician vs. head of department	0.18 (−0.080, 0.44)	0.19 (−0.096, 0.48); DF = 449
Students’ gender		
Male vs. female	−0.11 (−0.22, 0.001)	−0.051 (−0.18, 0.079); DF = 144
Students’ clinical skills (OSCE)		
Low vs. high performers	−0.15 (−0.35, 0.038)	−0.13 (−0.33, 0.078); DF = 139
Medium vs. high performers	−0.19** (−**0.33,** −**0.050)**	−0.18^*^(−**0.33,** −**0.038)**; DF = 141

Predictors remaining significant in the multifactorial model are reported in **bold**

*DF* degrees of freedom

*p < 0.05

**p < 0.01

***p < 0.001

Trainers were the most influential random effect in the multilevel model, with a covariance parameter estimate of 0.37, compared to 0.05 of the random effect of students and even lower estimates of the two other random effects (i.e. specialties and clinics). Moreover, the trainers’ random effect was nearly as large as the covariance parameter estimate of the residual (0.47).

## Discussion

In our analysis of a large sample of mini-CEX assessments in under-graduate medical education, mini-CEX scores could be predicted by OSCE scores only to a very limited degree. In contrast, characteristics of the context such as the trainers’ clinical position, clinic size and task complexity had a rather high impact on mini-CEX scores. As a guide to discuss these results, we will use the most important components of Govaerts’ model of clinical performance assessment [18], namely (1) the learners’ performance, (2) the raters, (3) the assessment design and (4) the organizational environment.

1. *The learners’ performance*. Students’ clinical skills as assessed in a standardized OSCE contributed little to the prediction of mini-CEX scores in the subsequent clerkships. At first glance, this is surprising, as assessments of clinical skills in standardized settings should help to predict ‘on-the-job-performance’ as reflected in mini-CEX [37, 38]. Indeed, there are several publications confirming the predictive validity of the OSCE [9–12]. However, due to ‘grade inflation’ and ‘range restriction’ in mini-CEX, as observed in many studies, both low and high OSCE performers in our study received almost identical ‘inflated’ mini-CEX scores, leading to weak correlations comparable to those found in other studies [14, 27, 39].
2. *The raters.* The trainers’ clinical position was the most important predictor of mini-CEX scores in our study. In line with other studies [14, 23], we found that residents gave the most lenient scores. This finding might represent residents’ self-perception as being learners themselves. In contrast, long-term experience in the use of WBA seems to foster the raters’ precision or stringency [33, 40].
  Compared to standardized OSCE settings, raters play a different role in the everyday clinical workplace application of WBA: Here, trainers often take more the role of mentors than raters [38]. High scores might be used to maintain the students’ motivation. Moreover trainers are reported to avoid ‘negative’ feedback [41], as students do not perceive feedback ‘free of emotions’, but may add a connotation such as ‘this person does not like me’ [42]. Thus, deterioration of the working relationship or reciprocally ‘negative’ clerkship evaluations might be anticipated by trainers as a consequence of critical feedback [24, 33, 43]. Accordingly, higher correlations between mini-CEX scores and assessments of clinical competence in post-graduate medical education were found when external examiners without a pre-existing relationship with the learner conducted the mini-CEX [44] or reviewed videotaped performance without personal interaction [45].
3. *The assessment design.* Our scale adaptations did not prevent score inflation, which corresponds with other studies: For example, several authors found high mean scores using the original 9-point mini-CEX scale [27, 46, 47], while others observed the same phenomenon e.g. using a 6-point scale [25, 48]. This illustrates that lenient ratings seem to be a common characteristic of numerical WBA scores in everyday clinical workplace applications―in contrast to judgments of video-taped performance [30].
  The complexity of the tasks also influenced mini-CEX scores in our study. Norcini already reported that trainers overcompensate their ratings for task complexity [8], which was also confirmed for the under-graduate setting [24]. Raters are often uncertain regarding which performance level to expect [24, 25]. In this situation, high scores might be assigned, and this was even more pronounced for difficult tasks in our study.
4. *The organizational environment*. Norcini and McKinley anticipated that WBA would not be equivalent across sites [38]. The clinic size indeed had an impact on scores in our study, as grade inflation was more pronounced in large clinics. Large university hospitals might have different workplace conditions compared to smaller affiliated hospitals [32]. This implies that priority for high-quality WBA might be even more limited in large clinics, contributing to inflated scores: It is less time-consuming for trainers to assign high scores, than to engage into sophisticated discussions about necessary improvements. On the other hand, personal relationships seem to be closer in smaller clinics, possibly enhancing accountability of raters and preventing excessive grade inflation [1]. Additionally, under less time pressure and based on closer relationships, students might feel more secure and more ready to accept even critical feedback [49].

### Strengths and limitations

This large sample of mini-CEXs included several specialties, clinics of different sizes and trainers with different clinical positions as well as multiple assessments per student. The multilevel design allowed us to include important organizational factors into the analysis and, for the first time, to determine their relative influence on WBAs precisely.

Limitations: OSCE scores themselves are not perfectly reliable, placing a limit on the possible correlation with other measures. However, even after a correction for attenuation, correlations between OSCE and mini-CEX scores would still be low, and would not markedly change our results as discussed above. The relative contribution of OSCE scores to predict mini-CEX scores is influenced by the way in which the variable is included in the model: We analyzed several variations, ranging from including OSCE scores as a continuous variable to using it as an ordinal variable with varying levels. None of these variations resulted in OSCE scores being a strong predictor for mini-CEX scores; its influence remained small compared to the remaining predictors in any model.

Additionally, one could argue that comparing a summative OSCE with formative WBA is similar to comparing ‘apples and oranges’. However, van der Vleuten et al. argue that summative and formative assessments represent a continuum rather than distinct categories [20]. Both forms of assessments should be reliable and valid to a certain degree (at least when several mini-CEXs are grouped together as in this study). Within a framework of ‘programmatic assessment’, each data point – either formative or summative – would meaningfully contribute to feedback and decision making [20]. As in practice, only averaged mini-CEX scores as well as averaged OSCE scores from different stations/encounters are used, e.g. for passing decisions, – while interpretation of single scores related to one station/encounter is not recommended – analyzing the relation between students’ mean OSCE scores and their mini-CEX scores in comparison with other influencing factors seemed justified. If both OSCEs and mini-CEXs are intended to measure clinical skills, they can be expected to be in line with each other.

As we retrospectively analyzed data occurring in a natural clerkship setting, the predictors represented predefined conditions of our clerkships. Our results could be used to include the most important factors into future studies and to vary them systematically within an experimental design. While these most important factors, trainer’s function and clinic size, could be well identified in this study, their precise functional relation remains vague, due to large variability in estimates.

### Implications for practice and research

In principle, WBA tools such as mini-CEXs have the potential to discriminate different levels of a learner’s performance. In the context of our study, low performing students were challenged by inconsistent information regarding their clinical skills: While the OSCE differentiates between different performance levels, in WBA, practically all students receive scores in the upper range of the scale. This is especially a problem for low performing students who might infer from inflated scores that there is little need for improvement.

Two options can be recommended as a consequence: to remove numerical scores from the forms and provide space for narrative feedback only―or to improve the design and implementation of rating scales in order to make scores more informative.

### Removing the scores

As numerical mini-CEX scores seem to have little informational value, some recent studies and recommendations concluded that they could be eliminated from the forms [50, 51]. Narrative feedback is rather suitable to inform learners regarding a change in practice compared to ‘tick boxes’ [52, 53].

### Improving WBA design and implementation

The validity of scores is threatened by many influencing factors in everyday clinical workplace performance assessment [24]. Evidence to support the value of training in WBA is conflicting [16, 54, 55]. As time for mini-CEX training always competes with time for patient management, we assume that trainers from our study were trained as extensive as possible within the clinical context. Nevertheless, some measures may be useful to improve WBA scorings: Reliability of scales and assessor discrimination can be enhanced if scales are ‘aligned to the construct of developing clinical sophistication and independence’ [2, 56], or in other words ‘entrustability’ [21, 57]. This implies that learning outcomes and relevant tasks (i.e. entrustable professional activities; EPAs [57, 58]) for each of the clerkships would be defined, facilitating ratings in relation to pre-defined expectations. Instead of merely placing emphasis on rater training, we should also consider Govaerts’ [18] recommendations for improvement of WBA assessment ‘by addressing organizational norms and values regarding performance assessment, transparency of assessment purposes and assessment process (due process), support, accountability and feelings of ‘psychological safety’ – based on open and honest communications and interactions between all stakeholders in the assessment process’.

## Conclusions

The results of our study indicate that mini-CEX scores might be more influenced by context characteristics than by the students’ clinical skills performance as assessed in a standardized setting. This adds to existing evidence that ‘the weakest component of the mini-CEX validity argument seems to be in the area of scoring’ [23]. Ways should be sought to either enhance the informational value of WBA scores or to abstain from them in favor of narrative comments only. This could help tapping the full potential of WBA―to prompt learning by means of supervision, feedback and reflection [59].

### Ethical approval

As only retrospective analyses of routinely collected, pseudonymised data were performed, the study was regarded exempt from ethical approval according to the Medical Faculty of the University of Bern. The study was carried out in accordance with the Declaration of Helsinki. The anonymity of the participants was guaranteed.

## Additional file

Additional file 1: **SAS statement and print-out of results.** (DOCX 985 kb)

## Abbreviations

WBA: workplace-based assessment
OSCE: objective structured clinical examination
Mini-CEXs: mini-clinical evaluation exercises
