# Supplementary material for: The influence of students’ prior clinical skills and context characteristics on mini-CEX scores in clerkships – a multilevel analysis
Source: BMC Med Educ. 2015 Nov 25;15:208. doi: 10.1186/s12909-015-0490-3 (PMC4658793; doi:10.1186/s12909-015-0490-3)

**Appendix – SAS statement and print-out of results**

**Article**: The influence of students’ prior clinical skills and context characteristics on mini-CEX scores in clerkships – A multilevel analysis

**Authors**:

Anja Rogausch (anja.rogausch@iml.unibe.ch)

Christine Beyeler (christine.beyeler@iml.unibe.ch)

Stephanie Montagne (stephanie.montagne@iml.unibe.ch)

Patrick Jucker-Kupper (patrick.jucker@iml.unibe.ch)

Christoph Berendonk (christoph.berendonk@iml.unibe.ch)

Sören Huwendiek (soeren.huwendiek@iml.unibe.ch)

Armin Gemperli (armin.gemperli@unilu.ch)

Wolfgang Himmel (whimmel@gwdg.de)

**SAS statement:**

ods graphics on;
proc mixed data=IML method=ml order=internal;
class specialty clinic expert_id student_id clinic size function student_gender complexity OSCE;
model mcex_overall =

clinic size function student_gender complexity OSCE / residual solution CL ddfm=kr;
random intercept / subject=specialty ;
random intercept / subject=clinic;
random intercept / subject=expert_id;
random student_id ;
lsmeans clinic size / cl;
lsmeans function / cl;
lsmeans student_gender / cl;
lsmeans complexity / cl;
lsmeans OSCE / cl;
run;
ods graphics off;

**Print-out of the results related to Table 2**. Estimated regression coefficients for the prediction of trainers’ ‘overall‘ mini-CEX scores, including random effects


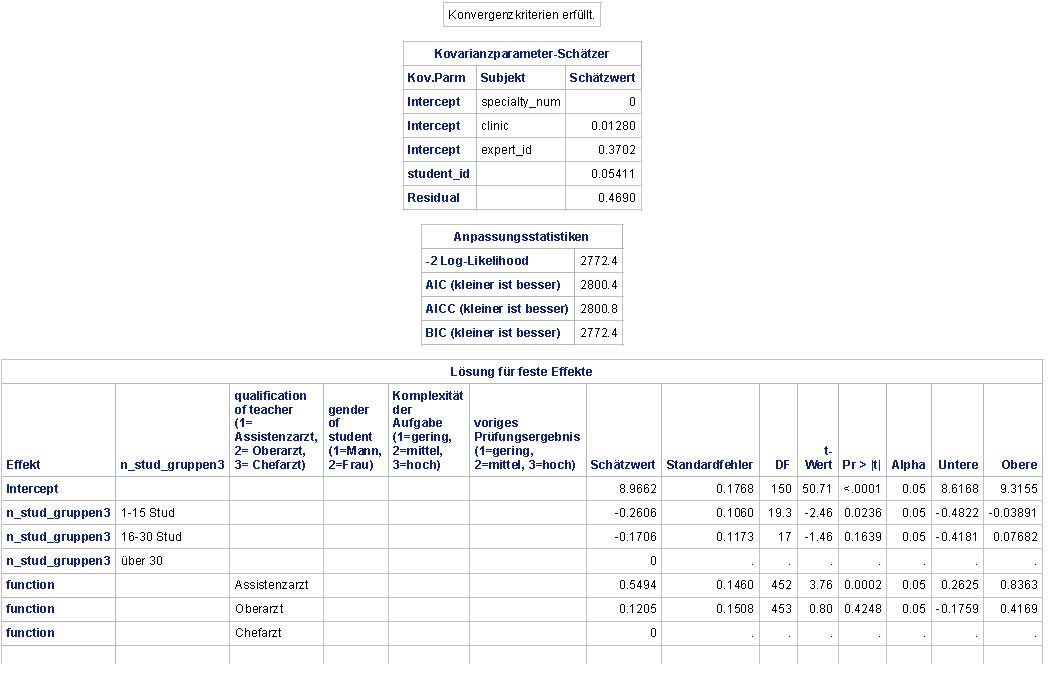

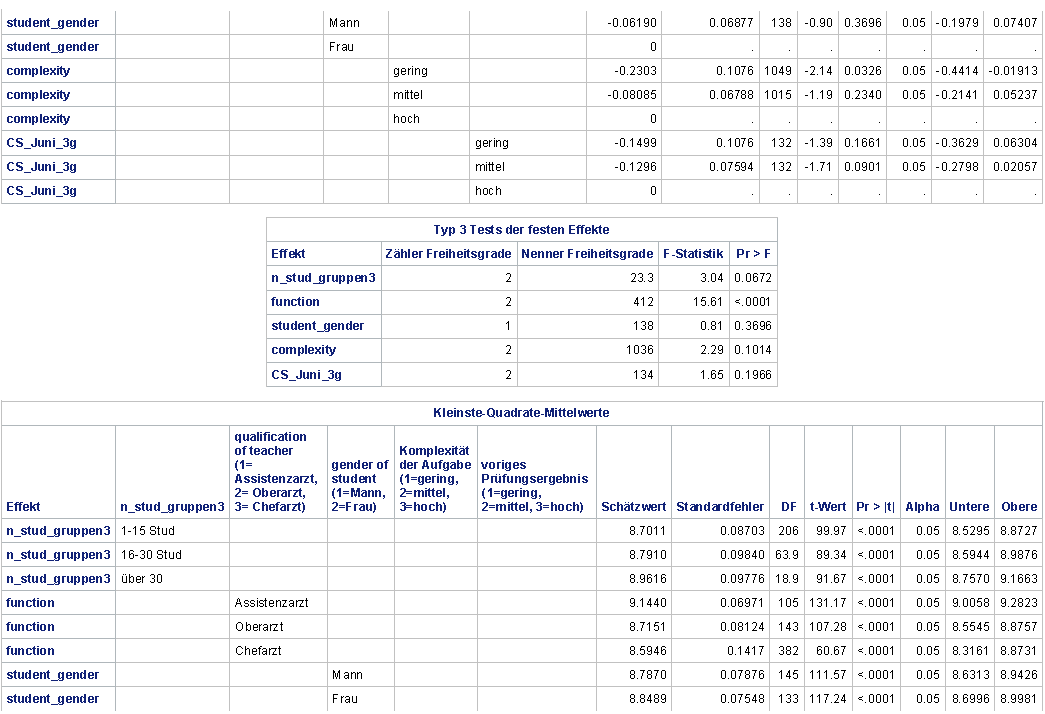


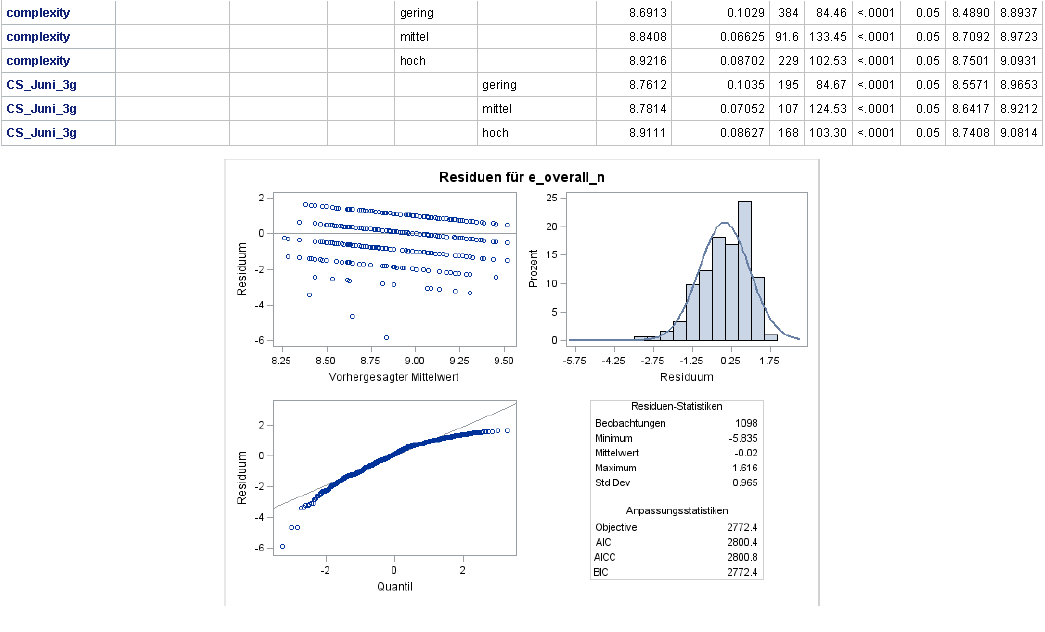

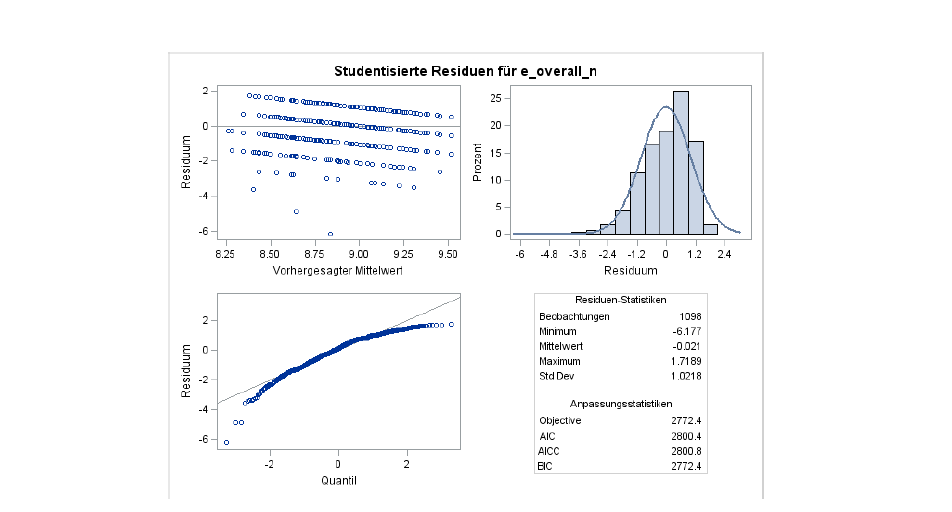


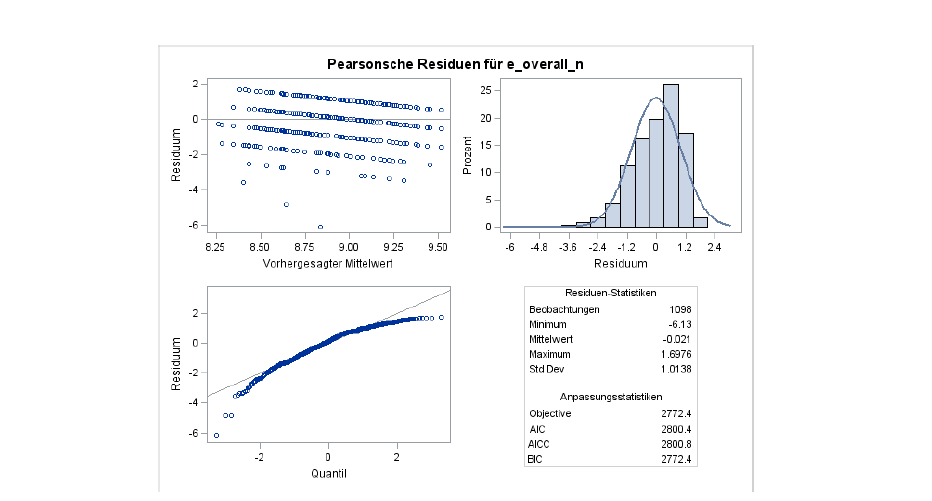


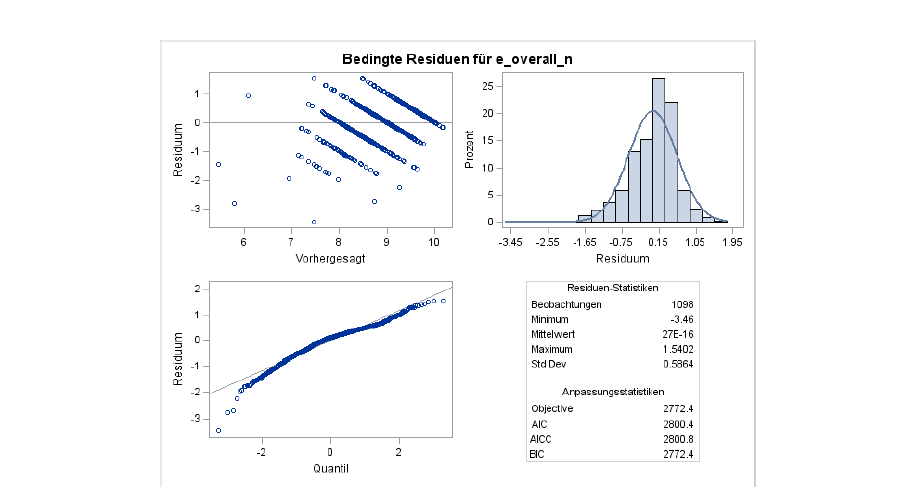


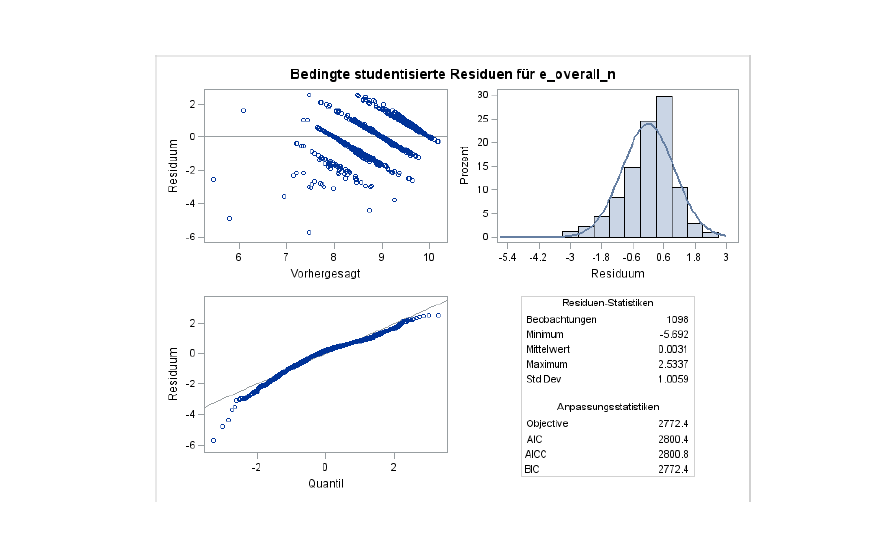

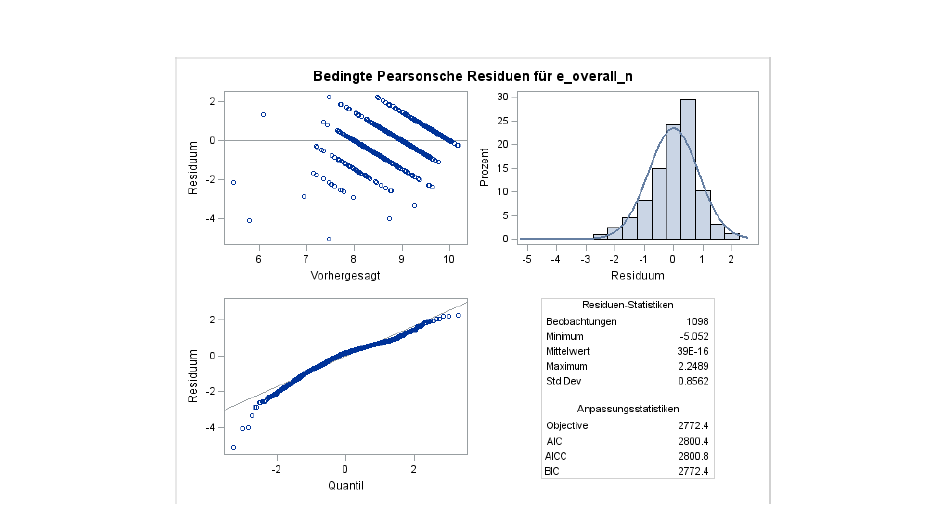


**Print-out of the results related to Table 3.** Estimated regression coefficients for the prediction of trainers’ mean ‘domain‘ mini-CEX scores, including random effects.


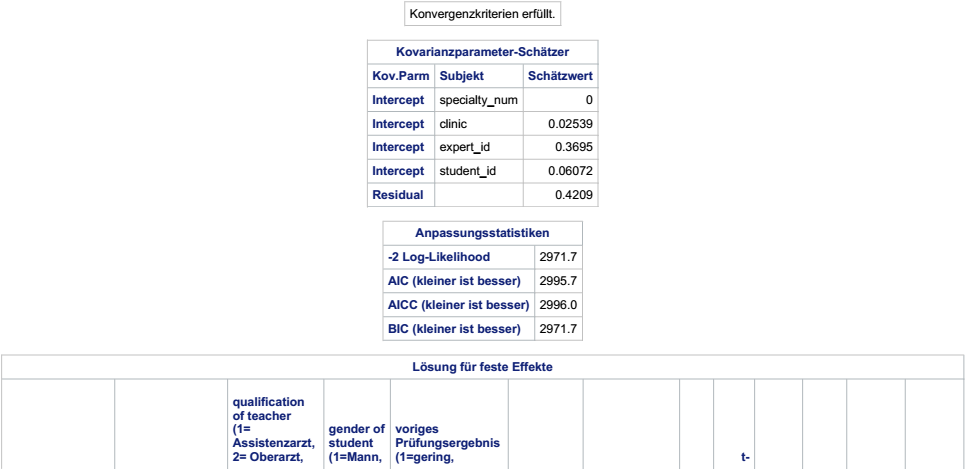

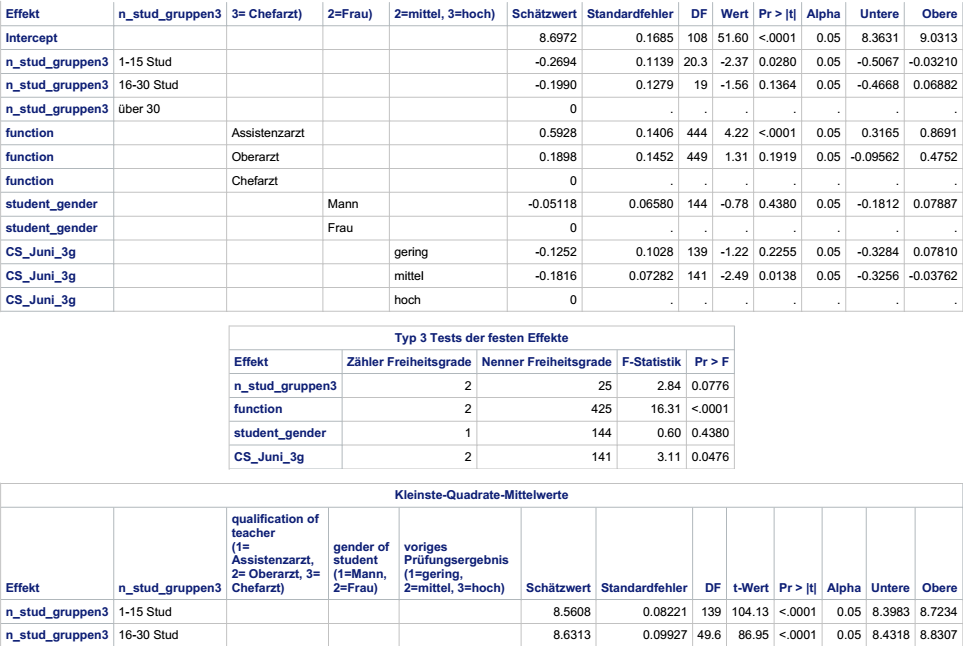

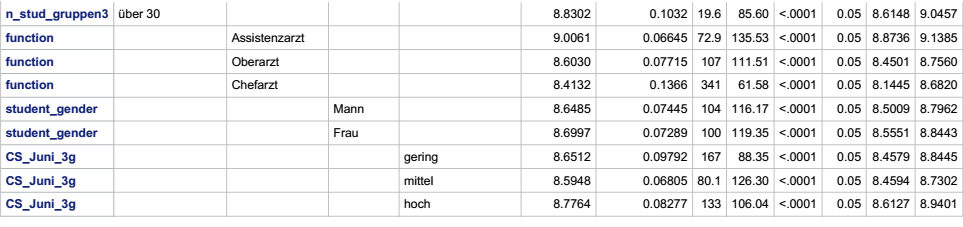


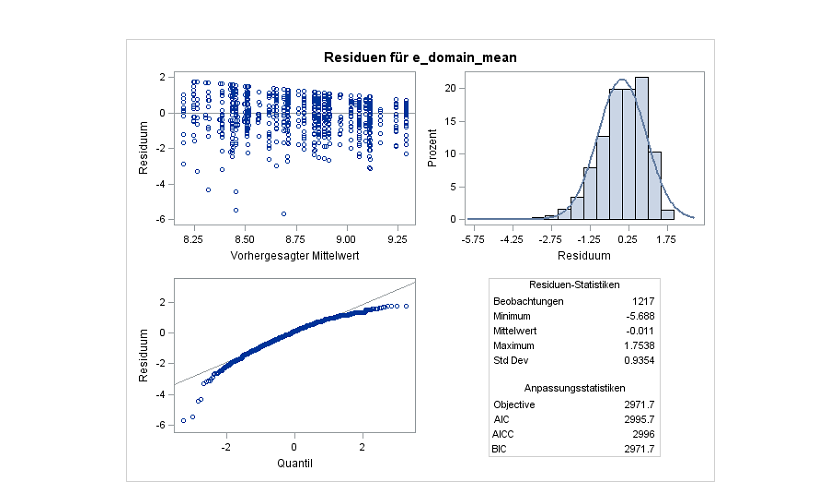


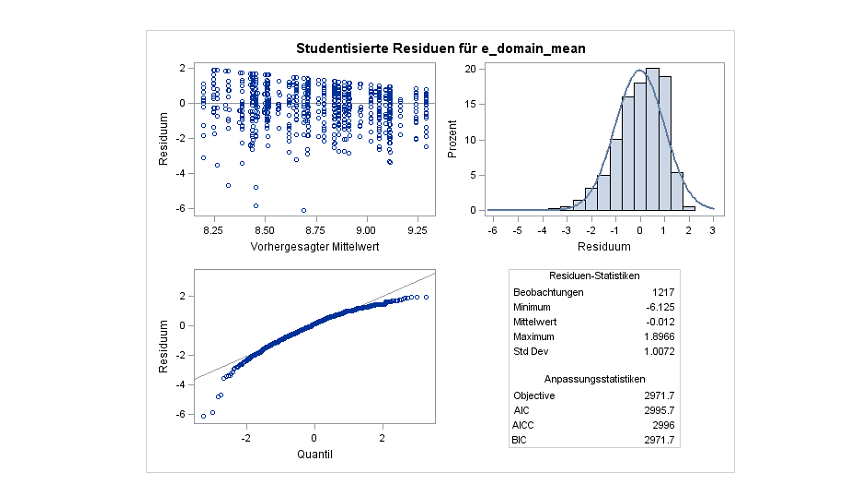


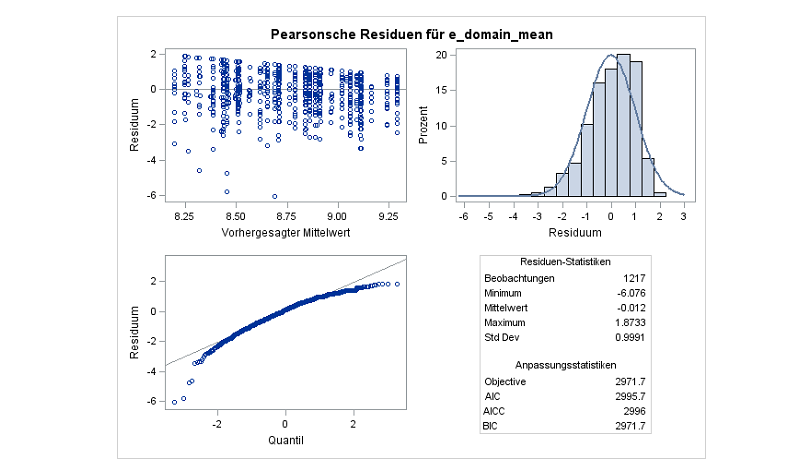

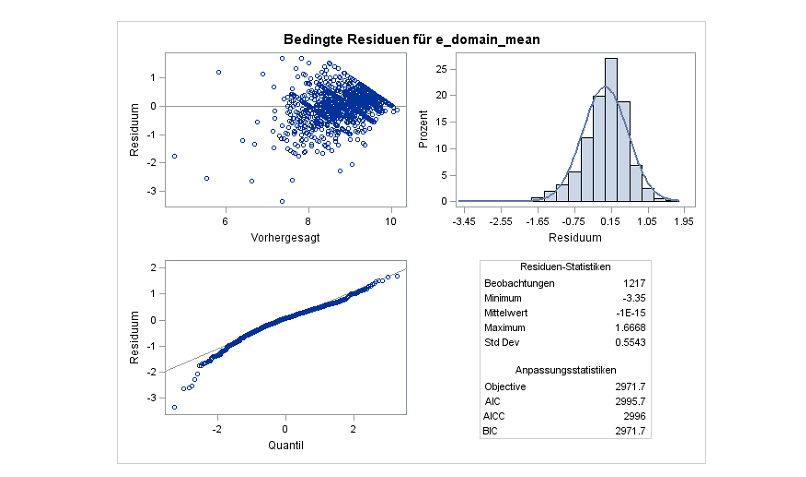

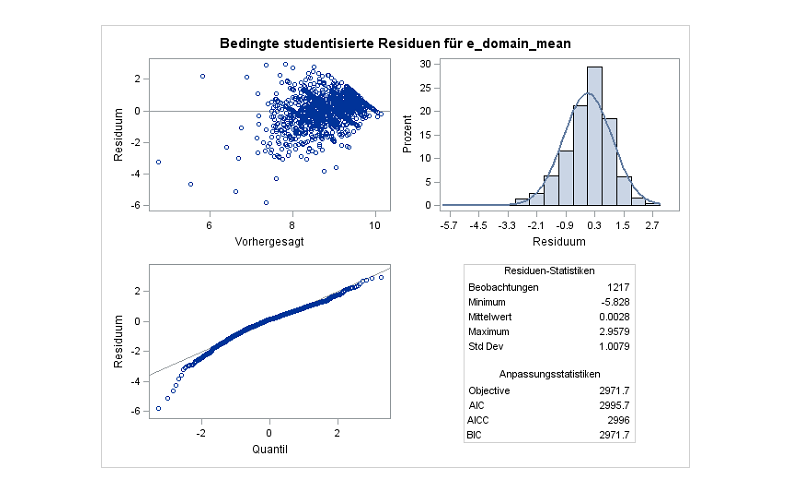


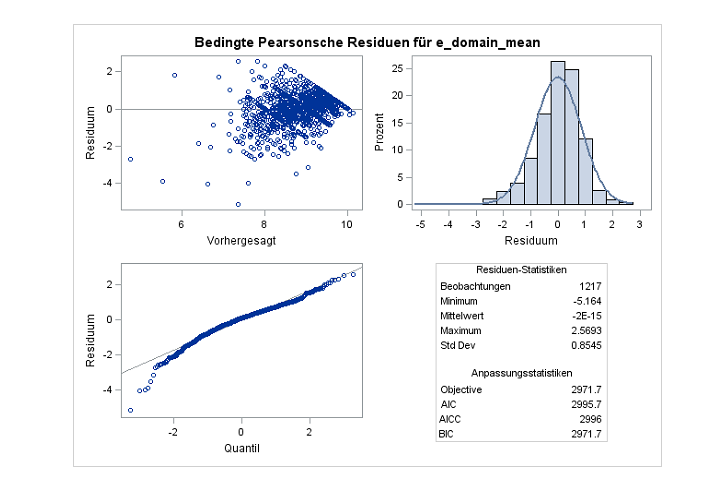

Supplement: Additional file 1: — SAS statement and print-out of results. (DOCX 985 kb) [file 12909_2015_490_MOESM1_ESM.docx]
